# Supplementary material for: A Comprehensive Overview of Baboon Phylogenetic History
Source: Genes (Basel). 2023 Feb 28;14(3):614. doi: 10.3390/genes14030614 (PMC10048742; doi:10.3390/genes14030614)
Supplement: Supplementary file 1 [file genes-14-00614-s001.zip › genes-2212916-supplementary.pdf]

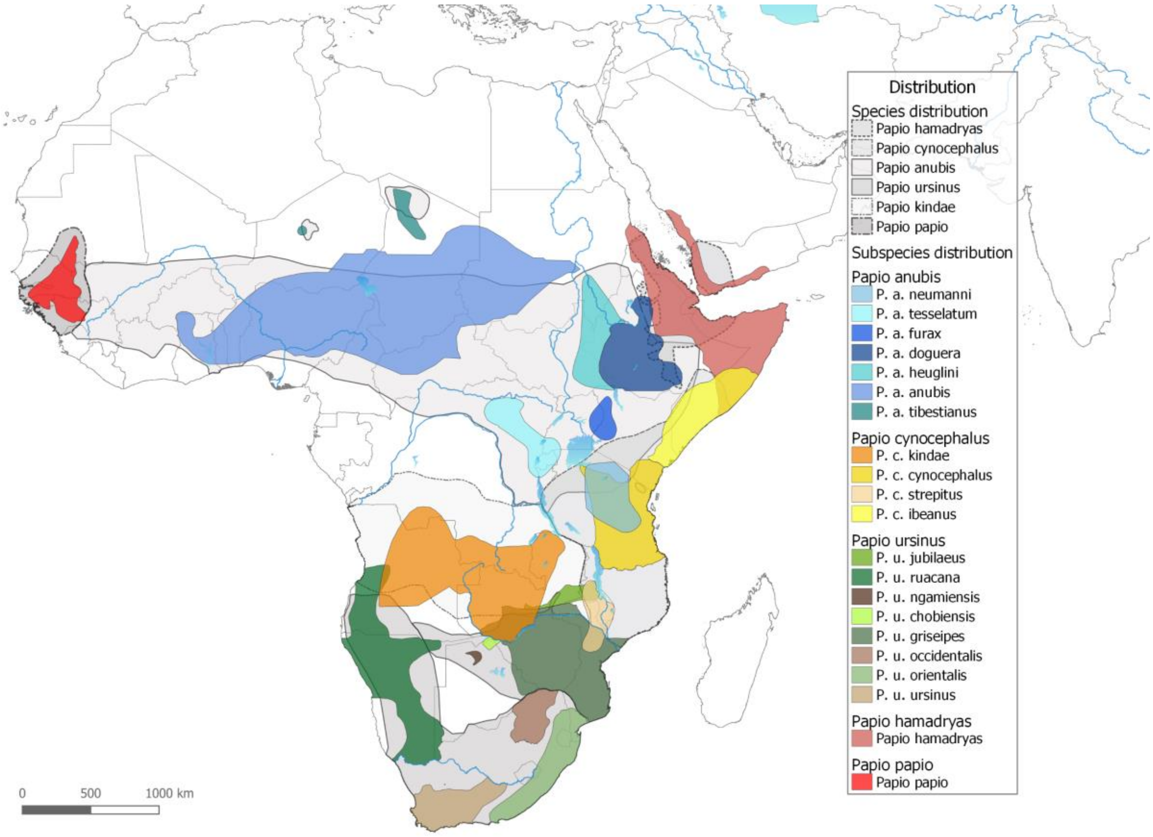

Figure S1: Distribution of baboon subspecies according to Hill (1970). Map created with QGIS v3.8, Species distribution shapefiles were obtained from IUCN red list (https://www.iucnredlist.org, last accessed: 15.10.20). Subspecies distribution was copied manually from Hill (1970). Country shapefiles were obtained from http://www.diva-gis.org/gData (accessed: 23.10.19) and river and lake shapefiles were obtained from WWF HydroSHEDS (https://hydrosheds.org/, accessed: 04.11.19).
